# Cancer biology: mechanism of antitumour action of vorinostat (suberoylanilide hydroxamic acid), a novel histone deacetylase inhibitor

**DOI:** 10.1038/sj.bjc.6603463

**Published:** 2006-12-12

**Authors:** V M Richon

**Affiliations:** 1 Merck Research Laboratories, Department of Cancer Biology and Therapeutics, BMB9-101, 33 Avenue Louis Pasteur, Boston, MA 02115, USA

**Keywords:** histone deacetylase, vorinostat, chromatin

## Abstract

Histone deacetylase (HDAC) inhibitors represent a potential new class of antitumor agents. Vorinostat (suberoylanilide hydroxamic acid or SAHA) is a potent inhibitor of HDAC activity and has undergone initial evaluation in several Phase I and II clinical trials. HDACs are enzymes that catalyse the removal of the acetyl moiety from the lysine residues of proteins, including the core nucleosomal histones. Together with histone acetyltransferases (HATs), HDACs regulate the level of protein acetylation. Alterations in both HAT and HDAC activity have been reported to occur in cancer. HAT activity has been found to be disrupted by translocation, amplification, overexpression or mutation in a variety of cancers, including those of haematological or epithelial origin. HDACs have been found to be overexpressed or associated with oncogenic transcription factors. Vorinostat induces growth arrest, differentiation or apoptosis in a variety of transformed cells. The antiproliferative effects of vorinostat are believed to be due to drug-induced accumulation of acetylated proteins, including the core nucleosomal histones and other proteins (e.g., BCL6, p53 and Hsp90). Phase I and II trials have been conducted for the oral formulations of vorinostat, and results show that vorinostat inhibits its target enzyme (HDAC) in peripheral mononuclear cells and tumour tissue at doses that are well tolerated. Antitumour activity has been seen in patients with both haematological and solid tumours.

Although a key characteristic of neoplastic transformation is unregulated cellular proliferation, cancer cells nevertheless retain their ability to undergo differentiation or apoptosis under certain circumstances (Marks *et al*, 2001). Research efforts have been focused on attempting to exploit this characteristic of tumour cells by identifying new anticancer therapies capable of triggering growth arrest, differentiation and/or apoptosis of transformed cells. In this regard, a new class of antitumour agents – histone deacetylase (HDAC) inhibitors – has received particular attention.

The HDAC inhibitor vorinostat (suberoylanilide hydroxamic acid or SAHA) blocks cancer cell proliferation both *in vitro* and *in vivo* with little or no toxicity to normal cells and has undergone evaluation in several Phase I and II clinical trials (Kelly *et al*, 2003, 2005; Duvic *et al*, 2005). This review discusses the effects of HDAC in modulating gene expression via histone acetylation and the role of abnormal HDAC activity in cancer development, summarises the wealth of *in vitro* and *in vivo* data demonstrating the antitumour effects of vorinostat, and explores current hypotheses on the potential mechanism(s) of action of vorinostat that may contribute to its antitumour activity.

## ROLE OF HDAC IN REGULATING GENE EXPRESSION AND CANCER DEVELOPMENT

Nucleosomes comprise the repeating unit of chromatin and serve to organise and compress the DNA in the nucleus. They are composed of the octamer of core histones (two molecules each of histones H2A, H2B, H3 and H4) spanning approximately 200 bp of DNA. The acetylation status of histones plays an important role in regulating gene expression by altering the structure of chromatin (Grunstein, 1997; Gregory *et al*, 2001). Regulation of histone acetylation is controlled by two enzyme families: histone acetyltransferases (HAT), which catalyses the addition of acetyl moieties to lysine residues of proteins, and histone deacetylases (HDAC), which catalyses their removal. HAT promotes gene transcription by acetylating histones, thereby facilitating an open chromatin structure. In contrast, HDACs deacetylate histones thereby facilitating a closed chromatin structure and hence transcriptional repression. Specifically, HDACs are believed to remove an acetyl group from the *ε*-amino group of the lysine side chain of histones H2A, H2B, H3 and H4, thereby reconstituting the positive charge on the lysine residues.

**Figure 1:**
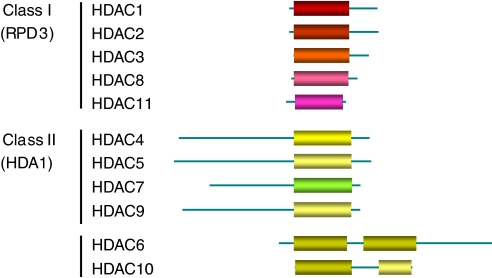
Classes I and II human HDACs categorised according to the homology of their catalytic domain and structure.

Three classes of HDAC have so far been identified: Classes I, II and III (for detailed review see Marks and Dokmanovic, 2005 and Verdin *et al*, 2003). The catalytic domain of Class I and II HDACs is NAD-independent and zinc-dependent, whereas the domain of Class III is NAD-dependent and zinc-independent. To date, a total of 11 Classes I and II human HDACs have been described, which are categorised according to the homology of their catalytic domain and structure (Figure 1). Class IIA enzymes have a long amino terminus and IIB enzymes have two catalytic domains. The different HDACs form large multiprotein complexes, including for example MTA2, Mi-2 and SMRT/N-CoR.

Alterations in the enzymes modifying histone acetylation are important from a cancer biology perspective in that HDAC is overexpressed in certain human cancers and is recruited by oncogenic transcription factors. For example, HDAC appears to be overexpressed in gastric (Song *et al*, 2005), prostate (Halkidou *et al*, 2004), and colon cancer (Zhu *et al*, 2004) and aberrant HDAC activity may also occur in certain forms of leukaemia (Fenrick and Hiebert, 1998) and lymphoma (Table 1) ([Lemercier *et al*, 2002). In acute promyelocytic leukaemia, for example, the transcriptional activator retinoic acid receptor alpha (RAR*α*) is fused with the promyelocytic leukaemia (PML) gene on chromosome 15 forming a complex (PML-RAR*α*) that results in HDAC recruitment and transcriptional repression (Grignani *et al*, 1998; He *et al*, 1998). Genes that encode HAT can also be translocated, amplified, overexpressed and/or mutated in various cancers, including haematological and epithelial malignancies (Table 1). One model of cancer formation, therefore, is the generation of deacetylated proteins due to the overactivity of HDAC or the inactivation of HAT.

## VORINOSTAT – A POTENT INHIBITOR OF HDAC ACTIVITY

Vorinostat (suberoylanilide hydroxamic acid or SAHA) is a nanomolar inhibitor of HDAC activity that has undergone initial evaluation in multiple Phase I and II clinical trials. Vorinostat is a small molecular weight (<300) linear hydroxamic acid compound that inhibits HDAC activity thereby inducing the accumulation of acetylated histones as well as nonhistone proteins, blocks the proliferation of cultured cells, and inhibits tumour growth in a variety of animal models. Vorinostat is a broad inhibitor of HDAC activity and inhibits both classes I and II enzymes (Marks *et al*, 2001;Marks and Dokmanovic, 2005). As with other HDAC inhibitors in clinical development, vorinostat does not inhibit HDACs belonging to Class III.

**Figure 2:**
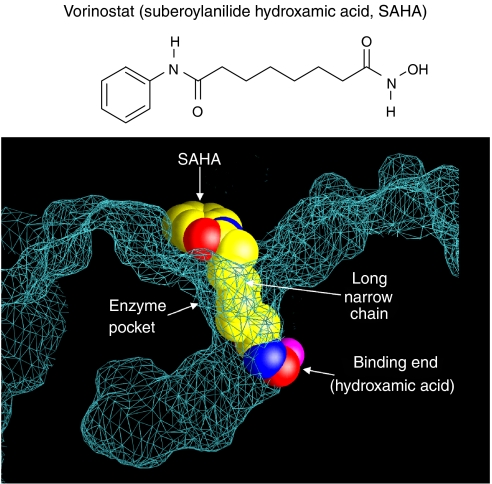
Vorinostat inhibits HDAC activity by binding to the pocket of the catalytic site. The hydroxamic acid moiety of vorinostat binds to a zinc atom (pink), allowing the rest of the molecule to lie along the surface of the HDLP protein (Marks *et al*, 2001).

Crystallographic studies have revealed that vorinostat inhibits HDAC activity by binding in the active site of the enzyme (Finnin *et al*, 1999). As shown in the molecular netting diagram (Figure 2), the hydroxamic end of the molecule binding to the zinc atom in the HDAC catalytic site, with the phenyl ring of vorinostat projecting out of the catalytic pocket on to the surface of HDAC.

## *IN VITRO* ANTITUMOR ACTIVITY OF VORINOSTAT

Vorinostat has been shown to inhibit the proliferation of a wide variety of transformed cells *in vitro*, including lymphoma, myeloma, leukaemia, and non-small cell lung carcinoma with concentrations that inhibit growth by 50% compared to no treatment ranging from approximately 0.5 to 10 *μ*M (Table 2) (Kelly *et al*, 2005). The inhibitory effects of vorinostat on cell proliferation tended to vary across multiple cell lines of a particular tumour type. This variability is illustrated by the recent findings from Koeffler and co-workers showing that vorinostat produced a profound but variable degree of inhibition of proliferation of lymphoma and leukaemia cells, including Burkitt, B-cell acute lymphoblastic leukaemia (B-ALL), MCL, DLBCL, ATL and T-cell (Sakajiri *et al*, 2005). For example, in the case of DLBCL, the ED_50_ for inhibition of cellular proliferation was 0.83 *μ*M for the SUDHL6 cell line and 1.9 *μ*M for the SUDHL16 cell line.

In addition to inhibiting the proliferation of transformed cells, vorinostat also inhibits proliferation of normal cells, as evidenced by comparing the effects of vorinostat on a matched panel of cells lines – normal human lung fibroblast cells (WI-38) and SV40 large T antigen transformed WI-38 (VA-13 cells) (Ungerstedt *et al*, 2005). Vorinostat resulted in a dose-dependent inhibition of proliferation of both cell types but was found to be selectively toxic to transformed cells, inducing death in tumour cells but leaving normal cells growth-inhibited but viable (Marks *et al*, 2001). The reason for this selective toxicity is incompletely understood, however, these studies provide evidence for an important role of thioredoxin in mediating the resistance of normal cells to vorinostat-induced cell death.

## *IN VIVO* ANTITUMOUR ACTIVITY OF VORINOSTAT

Vorinostat inhibits tumour growth in rodent models of a variety of solid tumours and haematological malignancies by both parenteral and oral administration (Table 3), including prostate cancer, (Butler *et al*, 2000) leukaemia (He *et al*, 2001), breast cancer (Cohen *et al*, 1999;Cohen *et al*, 2002), glioma (Eyupoglu *et al*, 2005) and lung cancer (Desai *et al*, 2003). Inhibition of tumour growth occurs at vorinostat doses that have little or no toxicity, as evaluated by weight gain, histological studies and gross anatomic examination of tissues/organs at autopsy (Butler *et al*, 2000). Vorinostat administered in the diet has been shown to inhibit the development of rat mammary tumours induced by the carcinogen *N*-methylnitrosourea (Cohen *et al*, 1999). Administration of vorinostat decreased tumour incidence and inhibited the growth of established mammary tumours (Cohen *et al*, 2002).

Vorinostat has also been shown to suppress growth of a transplanted androgen-dependent human prostate tumour (CWR222) in nude mice (Butler *et al*, 2000). At a dose of 50 mg kg^−1^ day^−1^, vorinostat decreased the mean final tumour volume by 97% compared with controls without detectable toxicity. Within 6 h of vorinostat administration, an increase in the accumulation of acetylated core histones was detected in CWR22 tumours.

On the basis of these and other studies, vorinostat has undergone evaluation in Phase I and II clinical trials in patients with solid tumours and haematological malignancies, including cutaneous T-cell lymphoma (Kelly *et al*, 2005, 2003; Duvic *et al*, 2005). These clinical studies are reviewed in detail in other articles comprising this supplement.

## MECHANISM OF ACTION OF VORINOSTAT

**Figure 3:**
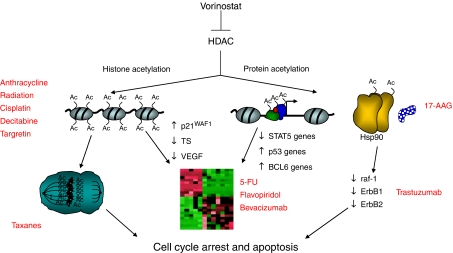
Proposed mechanisms of action of vorinostat in inducing tumor cell cycle arrest and apoptosis.

The mechanism for the antiproliferative effect of vorinostat is believed to be the result of inhibition of HDAC activity, resulting in the accumulation of acetylated proteins, including histones. Inhibition of HDAC activity by vorinostat has multiple cellular effects (Johnstone and Licht, 2003; Secrist *et al*, 2003). These effects include an alteration in the transcription of a finite number of genes (2–5% of expressed genes) via acetylation of histones and transcription factors, as well as nontranscriptional effects such as cell cycle arrest via inhibition of mitosis (Figure 3) (Marks *et al*, 2004). Vorinostat has been shown to impact the expression of several genes, some of which are induced (p21^WAF1^, TBP-2, gelsolin, metallothionein 1L, histone H2B) while others are repressed (cyclin D1, ErbB2, thymidylate synthase, importin b) (Glaser *et al*, 2003). Induction of gene expression for the cell kinase inhibitor p21^WAF1^ has been shown for several HDAC inhibitors including vorinostat and this effect may play a critical role in the growth arrest of transformed cells (Richon *et al*, 2000). Vorinostat induces up to a nine-fold increase in p21^WAF1^ mRNA and protein in T24 bladder carcinoma cells (Richon *et al*, 2000). This effect appears to be due in part to an increase in the rate of gene transcription associated with acetylation of the histones H3 and H4 associated with the p21^WAF1^ promoter (Richon *et al*, 2000; Gui *et al*, 2004).

Vorinostat may also promote the acetylation of numerous transcription factors, including androgen receptor, E2F-1, YY1, Smad7, EKLF, p53, BCL-6, HIF-1, NF-Y, NF-kappaB and GATA-1 (Marks *et al*, 2001; Secrist *et al*, 2003). Like histone acetylation, transcription factor acetylation may result in an alteration in the expression of certain genes. For example, acetylation of the p53 transcriptional activator leads to increased binding of p53 to DNA, which in turn, increases the expression of p53-regulated genes. Patients with lymphoma show increased activity of the transcriptional repressor BCL6, and acetylation of BCL6 can give rise to an inhibition of transcriptional repression by BCL6 (Bereshchenko *et al*, 2002).

In addition to histones and transcription factors, HDAC inhibitors have also been shown to acetylate the lysine residues of a number of proteins, including *α*-tubulin and the heat-shock protein Hsp90. Inhibition of HDAC6 activity leads to acetylation and disruption of Hsp90 and this, in turn, may lead to decreases in activity of progrowth and prosurvival client proteins, such as Bcr-Abl, mutant FLT-3, c-Raf and AKT in human leukaemia cells (Bali *et al*, 2005).

Evidence also exists that HDAC inhibitors influence the ability of tumour cells to undergo mitosis (Secrist *et al*, 2003). By causing an increase in acetylated histones and other proteins, HDAC inhibitors may disrupt the cell cycle and induce apoptosis of tumour cells by targeting cell cycle checkpoint controls – a G_2_ check point, which is often defective in tumour cells, and a mitotic spindle check point (Warrener *et al*, 2003). *In vitro* studies indicate that HDAC inhibitors give rise to aberrant spindles most likely by interfering with chromosome attachment, thereby producing mitotic accumulation without affecting mitotic microtubules (Sandor *et al*, 2000).

Further research into the mechanism(s) of action of vorinostat as well as delineation of its clinical utility in various cancer types should help formulate rational combinations with other chemotherapeutic agents that may provide synergistic or additive antitumour efficacy. Based on a mechanistic rationale, vorinostat has the potential to be combined with several different types of anticancer therapies, including radiation, anthracyclines, cisplatin, taxanes, 5-fluorouracil (5-FU), flavopiridol, bevacizumab and trastuzumab (Figure 3). Preliminary studies of tumour cells in culture suggest that vorinostat may be additive and even synergistic with radiation therapy and selected chemotherapeutic agents in inhibiting proliferation or inducing apoptosis (Table 4).

## SUMMARY

Vorinostat, a potent inhibitor of Classes I and II HDAC activity with an IC50 <86 nM, induces histone and protein acetylation and alters gene expression. Vorinostat blocks growth promoting signal transduction pathways and the proliferation of a broad spectrum of cultured cancer cells. Parenteral and oral administration of vorinostat at doses producing little or no toxicity to normal cells results in growth arrest in rodent models of several solid tumours and haematological malignancies, including prostate cancer, leukaemia, breast cancer, colon cancer and lung cancer. The mechanism underlying the antitumour action of vorinostat is not yet clear but may involve changes in the expression of specific genes via acetylation of histones and transcription factors as well as nontranscriptional effects such as inhibition of mitosis. Further research to delineate the mechanism(s) of action of vorinostat and other HDAC inhibitors may pave the way to developing rational combinations with other chemotherapeutic agents and perhaps ultimately to optimising chemotherapy regimens for cancer patients.

## Figures and Tables

**Table 1:** Summary of acetylation defects observed in various cancers

Leukemia	HAT fusions	HDAC mediated
	MOZ/CBP	PML/RARalpha
	MOZ/p300	PLZF/RARalpha
	MOZ/TIF-2	AML1 fusion
	MLL/CBP	
	MLL/p300	
Lymphoma		BCL6
		STAT5
		
Epithelial cancers	p300 mutations in colorectal, gastric, breast, pancreatic carcinomas/cancer cell lines	
	
Gastric cancer	Elevated expression of HDAC1	
	
Prostate cancer	Elevated expression of HDAC1	
	
Colon cancer	Elevated expression of HDAC1 and HDAC2	

**Table 2:** Vorinostat inhibits proliferation of a variety of transformed cells *in vitro*

Lymphoma	Breast adenocarcinoma
Myeloma	Pancreatic cancer
Leukaemia	Glioblastoma
Mesothelioma	Prostate cancer
Non-small cell lung carcinoma	Ovarian cancer
Bladder carcinoma	Melanoma
Colon carcinoma	Renal cell carcinoma
Thyroid cancer	Endometrial cancer

**Table 3:** Parenteral or oral administration of vorinostat inhibits growth of a variety of tumours in rodents

**Parenteral administration**	**Oral administration**
Prostate cancer (CWR22 xenograft)	Breast adenocarcinoma (*N*-methylnitrosourea-induced)
Leukaemia (acute promyelocytic leukaemia transgenic)	Lung tumours (tobacco-specific nitrosamine-induced)
Lymphoma (mantle cell, E*μ*-myc transgenic)	
Glioma (F98 cells)	

**Table 4:** Cell culture studies demonstrating that vorinostat enhances the antitumour efficacy of radiation therapy or chemotherapy

**Study**	**Combination**	**Cell lines**
Almenara *et al* (2002)	Vorinostat+flavopiridol	Leukaemia (U937)
Kim *et al* (2003)	Vorinostat+VP-16, ellipticine, doxorubicin, or cisplatin	Human glioblastoma (D54), breast (MCF-7)
Nimmanapalli *et al* (2003)	Vorinostat+imatinib	Chronic myelocytic leukaemia (LAMA-84)
Rahmani *et al* (2003)	Vorinostat+Hsp90 antagonist (17-allylamino-17-demethoxygeldanamycin)	Human leukaemia (U937).
Marchion *et al* (2004)	Vorinostat+topoisomerase II inhibitors	Breast
Rundall *et al* (2004)	Vorinostat+NF-kappaB inhibitor (BAY-11-7085)	NSCLC (A549, H157, H358, H460, H1299)
Chinnaiyan *et al* (2005)	Vorinostat+radiation	Prostate (DU145) and glioma (U373vIII)
Ocker *et al* (2005)	Vorinostat+5-FU+irinotecan	Hepatoma (HepG2, Hep1B and MH-7777A)
Rahmani *et al* (2005)	Vorinostat+perifosine	Leukaemia (U937, HL-60 and Jurka)
